# A new level of plasticity: *Drosophila* smooth-like testes muscles compensate failure of myoblast fusion

**DOI:** 10.1242/dev.126730

**Published:** 2016-01-15

**Authors:** Jessica Kuckwa, Katharina Fritzen, Detlev Buttgereit, Silke Rothenbusch-Fender, Renate Renkawitz-Pohl

**Affiliations:** Philipps-Universität Marburg, Fachbereich Biologie, Entwicklungsbiologie, Karl-von-Frisch Strasse 8, Marburg 35043, Germany

**Keywords:** Male fertility, Dumbfounded, Roughest, Sticks and stones, Hibris

## Abstract

The testis of *Drosophila* resembles an individual testis tubule of mammals. Both are surrounded by a sheath of smooth muscles, which in *Drosophila* are multinuclear and originate from a pool of myoblasts that are set aside in the embryo and accumulate on the genital disc later in development. These muscle stem cells start to differentiate early during metamorphosis and give rise to all muscles of the inner male reproductive system. Shortly before the genital disc and the developing testes connect, multinuclear nascent myotubes appear on the anterior tips of the seminal vesicles. Here, we show that adhesion molecules are distinctly localized on the seminal vesicles; founder cell (FC)-like myoblasts express Dumbfounded (Duf) and Roughest (Rst), and fusion-competent myoblast (FCM)-like cells mainly express Sticks and stones (Sns). The smooth but multinuclear myotubes of the testes arose by myoblast fusion. RNAi-mediated attenuation of Sns or both Duf and Rst severely reduced the number of nuclei in the testes muscles. Duf and Rst probably act independently in this context. Despite reduced fusion in all of these RNAi-treated animals, myotubes migrated onto the testes, testes were shaped and coiled, muscle filaments were arranged as in the wild type and spermatogenesis proceeded normally. Hence, the testes muscles compensate for fusion defects so that the myofibres encircling the adult testes are indistinguishable from those of the wild type and male fertility is guaranteed.

## Abstract (precis)

**Summary:** *Drosophila* testes muscles arise from stem cells and can compensate for fusion defects to safeguard fertility; this plasticity may compensate for the observed lack of satellite cells in *Drosophila*.

## INTRODUCTION

Function and formation of multinuclear myofibres are largely conserved within the animal kingdom. Numerous studies in vertebrates and *Drosophila melanogaster* have revealed that multinuclear striated myotubes arise by myoblast fusion (Abmayr and Pavlath, 2012). Despite the similarities, only vertebrates possess muscle stem cells, i.e. satellite cells, that allow growth and regeneration of muscles after injury (Wozniak et al., 2005). In *Drosophila*, the stem cells most similar to satellite cells are the adult muscle precursor cells (Figeac et al., 2007), which allow modification of larval muscles into templates for dorsal longitudinal indirect flight muscles during metamorphosis (Roy and VijayRaghavan, 1998). These stem cells are set aside during embryogenesis and amplify mitotically in the larvae (Bate et al., 1991). Most of them are associated with imaginal discs as adepithelial cells in late third instar larvae. During metamorphosis, they build the adult musculature of the legs, thorax, and female and male reproductive organs.

Muscle development in the *Drosophila* embryo has been studied intensively. In the embryo, heterotypic myoblasts recognize and adhere to each other. After signal transduction from the cell surface into the cell via adaptor proteins, F-actin reorganizes at the site of cell-cell contact, the opposing membranes are vesiculated and cytoplasmic continuity is established (Haralalka and Abmayr, 2010; Önel et al., 2014). In this process, several molecular players relevant for the formation of multinuclear myofibres have functional redundancies (Bonn et al., 2013; Duan et al., 2012; Hakeda-Suzuki et al., 2002; Hornbruch-Freitag et al., 2011). Well-studied examples of redundancy during myoblast fusion are cell adhesion molecules of the immunoglobulin superfamily (IgSF), namely Dumbfounded [Duf; also known as Kin of irre (Kirre)], and Roughest (Rst), which are expressed in founder cells (FCs) (Bate, 1990; Ruiz-Gómez et al., 2000). The genes encoding Duf and Rst are localized in the same region on the genome (St Pierre et al., 2014) and only the deletion of both leads to lack of fusion and embryonic lethality before sarcomere formation. Expression of Duf or Rst alone can rescue the deletion phenotype (Ruiz-Gómez et al., 2000; Strünkelnberg et al., 2001). Fusion-competent myoblasts (FCMs) express Sticks and stones (Sns) and Hibris (Hbs). Loss of Sns leads to a nearly complete block of fusion, whereas Hbs seems to be less essential (Bour et al., 2000; Dworak et al., 2001; Shelton et al., 2009).

**Fig. 1.**
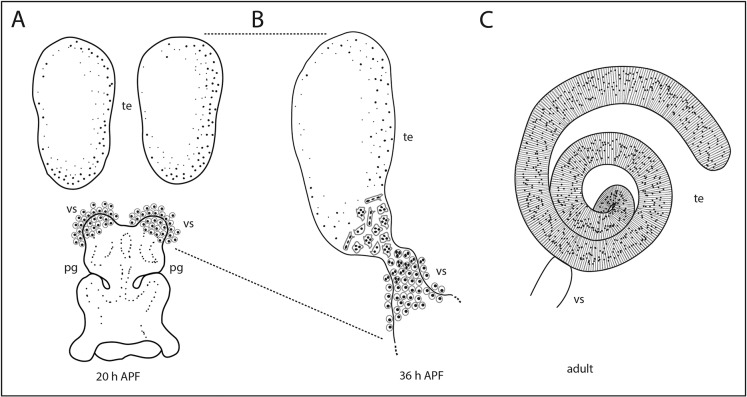
**Scheme of the origin and development of the testes muscles.** (A) Genital disc 20 h after puparium formation (APF) contains a pool of myoblasts on the protruding seminal vesicles (vs). The paired testes (te) are free of myoblasts. (B) By 36 h APF, the testis and the developing seminal vesicle are fused. Multinucleated nascent myotubes migrate onto the testis. (C) The adult testis is surrounded by a sheath of multinuclear smooth-like muscles. Modified after Bodenstein (1950); Susic-Jung et al. (2012).

All muscles of the *Drosophila* male reproductive system originate from adepithelial cells of the sexually dimorphic genital disc (Ahmad and Baker, 2002; Estrada et al., 2003; Kozopas et al., 1998). During metamorphosis, parts of the genital disc differentiate into the prospective seminal vesicle (vs) and the paragonia (pg) (Fig. 1A). The epithelial cells of the seminal vesicle and the developing testes connect to each other so that muscle precursors can migrate from the seminal vesicles onto the testes (Fig. 1B). Evidence from transplantation experiments and cultures of pupal testes indicates that the connection between the seminal vesicles and testes is essential for outgrowth and shaping of the testes (Gärtner et al., 2014; Kozopas et al., 1998; Nanda et al., 2009; Stern, 1941a,b). Different types of muscles can be found around the inner male genitalia, specifically multinuclear smooth-like myofibres surrounding the testes, multinuclear striated muscles of the sperm pump and a number of mononuclear striated muscles (Susic-Jung et al., 2012). In contrast to striated muscles, smooth muscles lack the regular arrangement in a repetitive pattern of sarcomeres with Z-discs and regular pattern of Myosins in the middle and F-actin linked to the Z-disc (Au, 2004). Smooth muscle cells are a heterogeneous group and they are well studied in mammals (Ali et al., 2005; Matsumoto and Nagayama, 2012). By contrast, all muscles of the female reproductive organs are mononuclear and striated (Hudson et al., 2008).

The molecular players responsible for the development of the muscles of the *Drosophila* male reproductive system and their mechanisms of action remain mostly unstudied. Likewise, little is known about the origin, development and function of mammalian peritubular myoid cells, i.e. the smooth muscle cells that enclose the seminiferous tubules of the testes where spermatozoa are generated (Svingen and Koopman, 2013). Recent analyses of the development of the different reproductive tract muscles of *Drosophila* have provided the first evidence that Duf, Sns and Hbs are relevant for arranging the multinuclear smooth-like muscles encircling the testes (Susic-Jung et al., 2012). In the present study, we focused on (1) how the multinuclear state of testes muscles is achieved; (ii) the role in this process of the IgSF proteins known from embryonic myogenesis (in particular, we asked whether these adhesion molecules act also redundantly during the development of the testes muscles); and (3) how loss of the adhesion molecules affects the formation of the male reproductive system and its musculature.

We show that the multinuclear smooth-like myofibres of the testes arise by fusion of two cell types resembling FCMs and FCs with respect to their heterotypic expression of IgSF proteins. All four IgSF proteins were involved in this process, although apparently with a function different to that in embryonic myoblast fusion. Importantly, even when fusion was reduced, the male reproductive tract developed as in the wild type. In this case, the smooth-like muscles displayed organized myofibres. Thus, our results reveal a high plasticity of smooth muscle formation.

## RESULTS

### Multinuclear myoblasts are found on the developing seminal vesicles of male genital discs

Myoblasts are characterized by the activity of the *Mef2* gene, which encodes the highly conserved muscle-specific transcription factor Mef2 (Bour et al., 1995; Lilly et al., 1994; Nguyen et al., 1994; Taylor et al., 1995). We established transgenic fly lines expressing *UAS-mCD8-GFP* under the control of *Mef2-Gal4* allowing ectopic expression in all myoblasts to follow myogenesis during metamorphosis with a live marker.

**Fig. 2.**
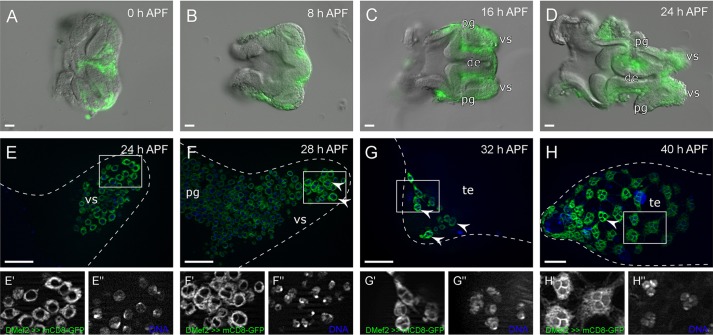
**Myoblasts on the male genital disc become multinuclear right before they migrate onto the testes.** (A) Myoblasts labelled by *Mef2*-driven *UAS-mCD8-GFP* expression (green) on male genital discs at the onset of puparium formation, (B) 8 h APF, (C) 16 h APF and (D) 24 h APF. (E) Additional Hoechst staining (blue) of DNA in the myoblast nuclei 24 h APF, (F) 28 h APF genital discs, and (G) 32 h and (H) 40 h testes. Single-channel magnifications of the boxed areas are displayed below. E,F,G and H, optical sections. Arrowheads, multinuclear myoblasts on the most anterior tips of the seminal vesicles or the testes. pg, paragonium; vs, seminal vesicle; de, ejaculatory duct; te, testis. Scale bars: 20 µm.

Myoblasts positive for *Mef2*-driven mCD8-GFP expression were present on the genital disc already at the onset of metamorphosis (Fig. 2A). During the first hours of metamorphosis, these myoblasts proliferated and increased in number [Fig. 1B,C, and monitored by anti-phosphorylated histone H3 (Ser10) mitosis marker staining in Fig. S1]. Myoblasts were found, 16 h after puparium formation (APF), in clusters on the prospective seminal vesicles, paragonia and ejaculatory duct (Fig. 2C). By 24 h APF, the primordia of the paragonia and the seminal vesicles were clearly distinguishable, and mCD8-GFP-positive myoblasts were particularly prominent on the seminal vesicles (Fig. 2D). These myoblasts will mainly give rise to the multinuclear smooth musculature of the testes and were still mononuclear at 24 h APF (Fig. 2E-E″). We first observed binuclear mCD8-GFP-positive myoblasts at 28 h APF, when the seminal vesicles grew closer towards the testes (Fig. 2F-F″). Here, mCD8-GFP expression is visible mainly in the cytoplasm and not efficiently integrated into the plasma membrane. These multinuclear myoblasts were restricted to the myoblast layer over the most anterior part of the prospective seminal vesicle (Fig. 2F, arrowheads); the paragonium still contained only mononuclear myoblasts. Up to this point, genital disc and testes are not attached and *Mef2*-positive cells cannot be found on the developing testes (Bodenstein, 1950; Susic-Jung et al., 2012). All *Mef2*-positive cells arriving on the testes at around 32 h APF are already multinuclear (Fig. 2G-G″). Those multinuclear mCD8-GFP-expressing cells cover the testes at 40 h APF (Fig. 2H-H″). Mononuclear *Mef2*-positive myoblasts were not observed on testes. These results showed that myoblasts amplify during metamorphosis and first become multinuclear on the prospective seminal vesicles of the male genital disc at around 28 h APF. We propose that further fusion leads to three, or rarely more, nuclei of the individual nascent myoblasts. This multinuclearity appears to be completed when the myotubes reach the testes.

### The multinuclear state of testes muscles is probably achieved by cell-cell fusion

To our knowledge, all multinuclear striated muscles in *Drosophila* arise by fusion of myoblasts. However, the other mechanism of generating multinuclear cells is by cytokinesis failure, i.e. nuclear division without cytokinesis. This process occurs in the early development of the *Drosophila* embryo and in mammalian heart muscles (Foe et al., 1993; Li et al., 1996; Liu et al., 2010). Thus, we investigated whether syncytial smooth-like muscles of the testes develop by cell cycle arrest after nuclear division. We labelled replicating DNA with the thymidine analogue 5-ethynyl-2′-deoxyuridine (EdU), which can be detected by covalent binding of a fluorescent azide.

**Fig. 3.**
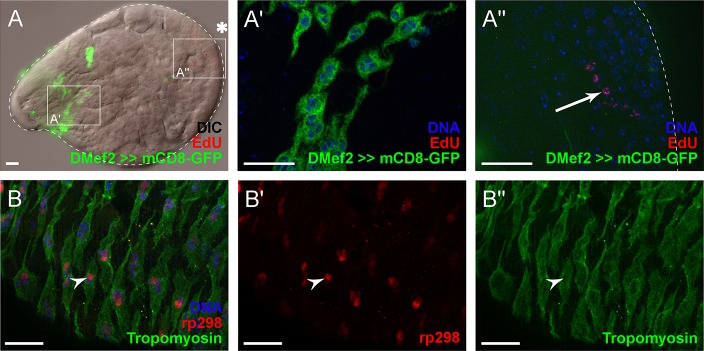
**Testes myoblasts fuse to generate multinuclear myotubes.** (A-A″) Labelling of replicating DNA with 5-ethynyl-2′-deoxyuridine (EdU, red; arrow in A″) in pupal testis at 24 h APF, followed by incubation for 8 h. Myoblasts were labelled by *Mef2*-driven *UAS-mCD8-GFP* expression (green). Asterisk, hub region; magnified areas shown in A′ and A″ are indicated in A. Additional Hoechst staining (blue) of DNA (A′) in the basal area and (A″) in the apical part of the testis. (B-B″) Visualization of *rp298-lacZ* (red) in nascent myotubes (green) in pupal testes at 40 h APF; B is a merged photo of B′ and B″ with additional Hoechst staining. Arrowhead, *rp298-lacZ* positive nucleus. A′,A″ and B-B″, optical sections. Scale bars: 20 µm.

Male pupae with *Mef2*-driven mCD8-GFP were injected with EdU at 24 h APF, shortly before the first binuclear myoblasts formed. After incubation for 8 h, the pupae were dissected and fixed, EdU and GFP fluorescent labels were analysed. During the 8 h incubation, myoblasts became multinuclear and migrated from the male genital disc onto the testis. On the testis, myoblasts expressing mCD8-GFP were detected in the basal area (Fig. 3A, left). At higher magnification, no EdU signal was detected in the nuclei of these nascent myotubes (Fig. 3A′). By contrast, germ cells in the apical part of the testis (Fig. 3A, right) were clearly labelled with EdU (Fig. 3A″, arrow). This demonstrated the progression of development, as well as successful incorporation and detection of EdU in dividing germ cells as an intrinsic positive control. On the basis of these results, we concluded that the multinuclear state of testes muscles arises in a cell-cycle-independent manner.

To further investigate how the multinuclear testes muscles are formed, we analysed the expression pattern of the *rp298-lacZ* enhancer trap (Nose et al., 1998). This transgene follows the activity of the *duf* enhancer and is located to the nucleus by the NLS of the transposase (Mlodzik and Hiromi, 1992). In the *Drosophila* embryo, *rp298-lacZ* can be found in nuclei of FCs, but not in FCMs. After fusion, all nuclei in the body wall muscles are positive for *rp298-lacZ* (Nose et al., 1998). By contrast, multinuclear myotubes of testes at 40 h APF expressed *rp298-lacZ* predominantly in only one nucleus (Fig. 3B-B″, arrowhead), as has been described for binuclear circular visceral muscles (Klapper et al., 2002). This finding suggested that the nuclei of one testis muscle originate from different myoblasts.

Taken together, these data indicated that nuclear division with cytokinesis failure does not occur during the development of *Drosophila* testes muscles. We thus concluded that a myoblast fusion process is obligatory in this context and therefore investigated whether testes myoblast fusion is comparable to the FC- and FCM-based mechanism by which striated muscles are established in *Drosophila*.

### Myoblasts on the prospective seminal vesicles show differences in IgSF expression

**Fig. 4.**
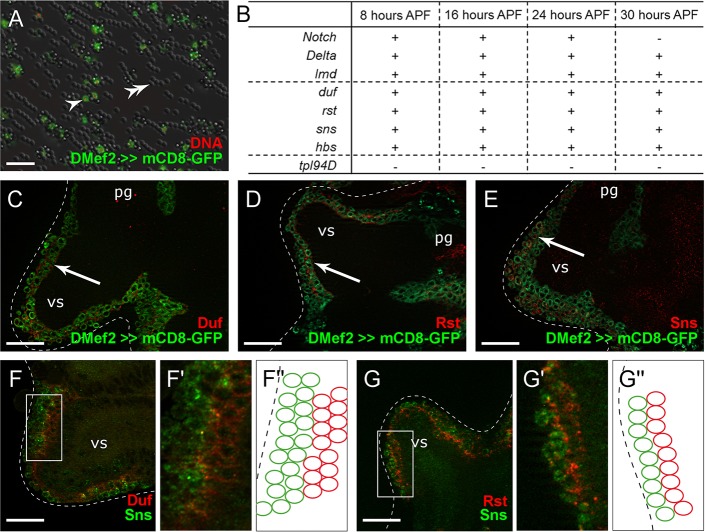
**Adhesion molecules are transcribed and expressed in myoblasts on the male genital disc.** (A) Myoblasts expressing *Mef2*-driven *UAS-mCD8-GFP* (green, arrowhead) purified using magnetic beads (double arrowhead). Hoechst staining of DNA (red). (B) RT-PCR results using RNA from purified myoblasts at 8 to 24 h APF. (C-E) Staining in male genital discs at 24 h APF in a *Mef2*-driven *UAS-mCD8-GFP* background with antibodies against (C) Duf, (D) Rst and (E) Sns. Arrows, myoblasts located on the prospective seminal vesicles. (F-G″) Double staining in male genital discs at 24 h APF with antibodies against (F,F′) Sns and Duf and (G,G′) Sns and Rst. F′ and G′ are enlargements of boxed areas shown in F and G, respectively. F″ and G″ are schematic depictions of the antibody distribution seen in F′ and G′. C-G′, optical sections. vs, seminal vesicle; pg, paragonium. Scale bars: 20 µm.

To gain insights into the molecular players involved in testes myoblast fusion, we checked for the presence of transcripts of known myogenesis-relevant genes in the myoblasts of male genital discs at different time points during metamorphosis. We established a protocol to purify myoblasts from male genital discs. Total RNA of age-specific mCD8-GFP-positive myoblasts was isolated (Fig. 4A) and used for RT-PCR analyses (Fig. 4B, Table S1). Hoechst staining of DNA revealed the high purity of isolated myoblasts (Fig. 4A).

Initially, we investigated whether distinct myoblasts similar to embryonic FCMs and FCs exist on the genital disc. In the embryo, Notch/Delta-mediated lateral inhibition leads to specification of FCs and FCMs (Baker and Schubiger, 1996; Carmena et al., 1995; Corbin et al., 1991). We confirmed active transcription of the gene encoding the Notch receptor and the gene encoding its ligand Delta in purified myoblasts (Fig. 4B), and lack of transcription of the spermatid specifically expressed negative control *Transition protein like^94D^* (*Tpl^94D^*) (Rathke et al., 2007). We also demonstrated transcription of the FCM-specific determination factor *lame duck (lmd)* (Duan et al., 2001) and FC-specific transcription factors (Fig. 4B, Table S1). Several, but not all, genes encoding transcriptional regulators conferring FC or muscle identity in the embryo were transcribed in these myoblasts (Table S1). Thus, we hypothesize that there are FC- and FCM-like myoblasts on male genital discs.

We further investigated whether myoblasts express the genes encoding IgSF proteins that initiate embryonic myoblast fusion. Using gene-specific oligonucleotides, we were able to amplify the adhesion molecule transcripts *duf*, *rst*, *sns* and *hbs* from myoblast RNA of genital discs 8, 16, 24 and 30 h APF (Fig. 4B), i.e. not only during fusion events, but also earlier. However, this method cannot distinguish between transcripts from testes myoblasts and transcripts from myoblasts that give rise to other muscles of the reproductive tract, such as multinuclear striated muscles of the sperm pump. Therefore, to determine the precise expression pattern and the subcellular localization of these IgSF proteins, we used immunofluorescence staining. In the prospective seminal vesicles of genital discs at 24 h APF, anti-Duf (Fig. 4C), anti-Rst (Fig. 4D) and anti-Sns (Fig. 4E) signals were detected in the membranes of *Mef2*-positive myoblasts (arrows). The adhesion molecules were distributed evenly over the plasma membrane and not localized to specific regions; as mCD8-GFP expression was localized further into the myoblast than IgSFs, we assume that mCD8 is mainly cytoplasmic in these cells (see also Fig. 2). Only a subset of myoblasts was stained with each antibody; Duf and Rst appeared to be enriched in myoblasts adjacent to the epithelium of the primordial seminal vesicles (Fig. 4C,D), whereas Sns accumulated in myoblasts lying on the periphery of the tissue (Fig. 4E). Furthermore, we did not observe colocalization of Sns and Duf (Fig. 4F,F′) or of Sns and Rst (Fig. 4G,G′) in double staining, which indicated expression of Sns in myoblasts other than those that expressed Duf and Rst. We hypothesize that these different subsets of myoblasts fuse to each other to generate smooth-like testes muscles. Consequently, we refer to them as FC-like myoblasts and FCM-like cells. FC-like myoblasts express Duf and Rst, whereas FCM-like cells mainly express Sns.

### Knockdown of Duf, Rst or Sns leads to a lower nuclei number in testes muscles

As the adhesion molecules Duf, Rst and Sns were expressed, we analysed whether their presence is essential for the formation of multinuclear testes muscles. As *hbs* transcript could be detected in myoblasts isolated from the genital disc, we included *hbs* in this functional study. We down-regulated *duf*, *rst*, *sns* and *hbs* specifically in myoblasts using *Mef2-Gal4*-driven RNA interference (RNAi). The adult-muscle-specific driver line 1151-Gal4 did not reveal any activity in the myoblasts of the male genital disc and was therefore excluded from further studies (data not shown). Because Duf and Rst can act in redundancy to each other, we additionally downregulated both *duf* and *rst* in a *duf-RNAi;rst-RNAi* double knockdown. For comparison, we expressed *Dicer-2* (*Dcr-2*) in the RNAi background, because it enhances the effect of RNAi (Dietzl et al., 2007).

**Fig. 5.**
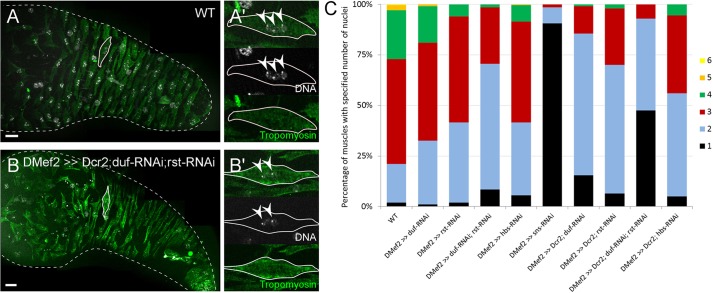
**Knockdown of adhesion molecules leads to reduced nuclei number in testes muscles.** (A,B) Visualization of muscles and nuclei using anti-Tropomyosin (green) and Hoechst staining (white) of (A) wild-type myotubes and (B) myotubes expressing *Mef2*-driven *Dcr-2*, *duf-RNAi; rst-RNAi*. Images are merged photos of single optical sections. The outlined muscle is displayed in split channels in A′,B′. Arrowheads, muscle nuclei. Scale bars: 20 µm. (C) Determination of nuclei number of nascent myotubes in which *duf*, *rst*, *duf;rst*, *hbs* and *sns* were downregulated by *Mef2*-driven RNAi and in wild-type testes at about 42 h APF. In each case, 200 muscles (100% of the cells) were counted; the length of each colour-coded bar in each stack indicates the percentage of muscles with the specified number of nuclei (see Table S2 for details).

As the muscle sheath tightly encircles the adult testis, single muscles and their cell nuclei are difficult to visualize; we saw only 1-2 nuclei on one side of the testes (Susic-Jung et al., 2012; Fig. 1C). First nascent myotubes reach the testes at 32 h APF (Fig. 2G) and are still individually distinguishable at 48 h APF; they did not surround the testes at this time (Fig. 2H, 40 h APF). At these time points, only multinucleated nascent myotubes but no mononuclear myoblasts were observed on the testes (Fig. 2E,F). We counted the number of nuclei in nascent myotubes on wild-type testes at 36 h, 42 h and 48 h APF (Table S2). The number of nuclei did not increase during ongoing metamorphosis, making further fusion on the testes unlikely. We conclude that fusion mainly takes place on the genital disc. Therefore, we analysed testes from pupae around 42 h APF, because migrating nascent myotubes have not stretched to their full size, which allows individual myotubes to be clearly recognized and the number of nuclei per muscle to be easily determined (Fig. 5A,B).

In the wild type, the majority of nascent myotubes comprised 3 nuclei, and some had 2 or 4 nuclei (Fig. 5C); the average was 3.1 nuclei. In rare cases, mononuclear myotubes or myotubes with 5 or 6 nuclei were observed. In myotubes in which *duf*, *rst*, *hbs* or *sns* were individually downregulated, the number of nuclei was reduced (Fig. 5C, see also Figs S2, S3 and Table S2 for controls and details). Co-expression of *Dcr-2* with the RNAi constructs led to a more severe reduction in nuclei number (Fig. 5C); expression of either *duf-RNAi* or *rst-RNAi* with *Dcr-2* increased the number of mono- and binuclear myotubes from the observed 21% in the wild type to 85% and 70%, respectively. The average number of nuclei declined to 2 and 2.3, respectively. Knockdown of *hbs* resulted in an average number of 2.4 nuclei, which was slightly lower than that of the controls. When both *duf* and *rst* were downregulated, the number of nuclei was reduced further than in the single knockdowns. Again, co-expression of *Dcr-2* led to a more severe reduction in the nuclei number; mono- and binuclear muscles were observed in 93% of the myotubes, with an average nuclei count of about 1.6. Expression of a *sns*-*RNAi* construct by *Mef2*-*Gal4* led to the most severe reduction in nuclei, with an average of about 1.1; more than 90% of myotubes remained mononuclear. Furthermore, these pupae did not develop into adults. Co-expression of *Dcr-2* with *sns-RNAi* resulted in early pupal lethality and therefore could not be analysed. From these findings, we concluded that the number of nuclei in testis muscles depends on the IgSF proteins that initiate myoblast fusion in the embryo.

### Development of the male reproductive tract and spermatogenesis appears normal despite a severe reduction in fusion efficiency

We then analysed whether the nuclei number of testes muscles affects functionality. We tested whether males in which *duf*, *rst* or *hbs* were downregulated were fertile. Three virgin wild-type females were mated with single males for 7 days; we tested up to 30 RNAi knockdown males. Offspring were evaluated 14 days after mating started. *sns*-*RNAi* males could not be analysed because depletion of *sns* led to pupal lethality.

**Fig. 6.**
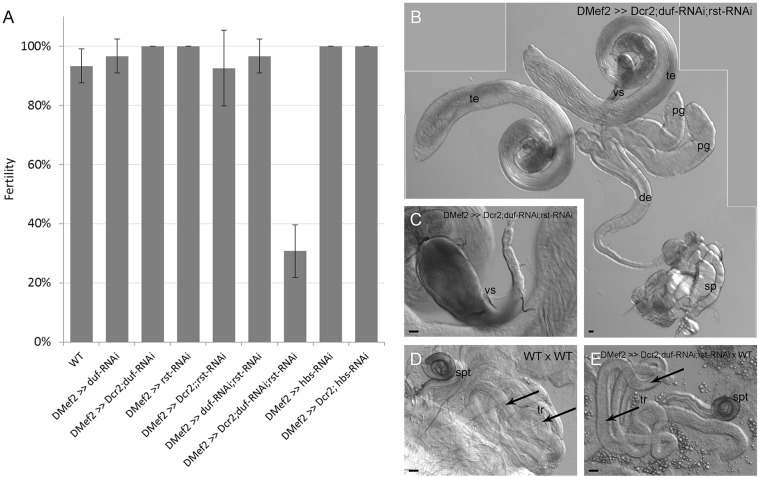
**RNAi-mediated knockdown of *duf*, *rst* or *hbs* in the reproductive tract musculature does not influence male fertility.** (A) Fertility of *duf*, *rst*, *duf;rst* and *hbs* knockdown males compared with wild-type males. Error bars represent s.d. (B) Reproductive tracts of infertile males (merged photograph). (C) Seminal vesicles of infertile males are filled with mature sperm. (D) Wild-type females mated with wild-type males contain sperm in their tubular receptacles (arrows) or spermatheca. (E) Reproductive tracts of females of infertile crossings with RNAi knockdown males are free of sperm (arrows). spt, spermatheca; tr, tubular receptacle; te, testis; vs, seminal vesicle; pg, paragonium; de, ejaculatory duct; sp, sperm pump. Scale bars: 10 µm in B, 20 µm in C-E.

Males with downregulated *duf*, *rst* or *hbs*, alone or in combination with *Dcr-2* as well as the *duf;rst* double knockdown produced offspring, which indicated normal fertility. Only males with *Mef2*-driven *duf;rst* double knockdown in the *Dcr-2* background had reduced fertility (Fig. 6A). Only 31% of single crossings produced offspring, and the number of offspring was lower than in the other males (Table S2 and Fig. S4); 69% were infertile. The reproductive tract of these infertile males had no visible blockages, constrictions or other defects (Fig. 6B). The seminal vesicles were filled with motile sperm (Fig. 6C, Movie 1); no obvious defects in spermatogenesis were detected. To identify the cause of reduced fertility in these flies, we first checked the mated wild-type females that failed to produce progeny. In wild-type female reproductive organs, sperm are stored in the spermatheca (spt) and the tubular receptacles (tr; Fig. 5D). The spermatheca and tubular receptacles of females mated with RNAi knockdown males were free of sperm (compare Fig. 6D and E). Subsequently, we examined the fertility of *Mef2*-driven *Dcr-2;duf-RNAi;rst-RNAi* females by crossing them with wild-type males. Their fertility was also severely reduced, showing that the effect was not sex-specific as would be expected for defects due to muscles of the testes. Furthermore, these males and females were able to crawl and jump but were unable to fly (Table S2). As sperm production in the males is not disturbed and females display reduced fertility, we assume that the cause of reduced fertility in these flies is a general problem in behaviour and/or movement rather than defective testes muscle development. We also conclude that except for *Dcr-2;duf-RNAi;rst-RNAi*, a reduced number of nuclei in the testes muscles does not necessarily interfere with male fertility.

### Knockdown of *duf*, *rst* or *hbs* leads to a wild-type adult muscle pattern with a normal filament arrangement

**Fig. 7.**
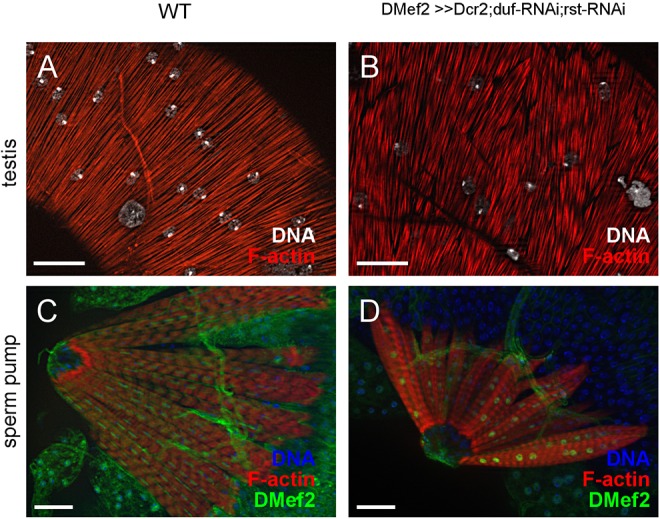
**Knockdown of *duf* and *rst* during development does not lead to defects in filament organization in adult reproductive muscles.** Filament arrangement of (A,C) wild-type and (B,D) *duf-RNAi;rst-RNAi* double knockdown in (A,B) smooth-like testes muscles and sarcomere pattern of (C,D) multinuclear sperm pump muscles. F-Actin (red) was visualized with Phalloidin; Hoechst dye (white in A and B; blue in C and D) was used to label DNA in nuclei. In C and D, anti-Mef2 antibody (green) was used to detect muscle nuclei. All photographs are optical sections. Scale bars: 20 µm.

As fertility was not affected by RNAi-mediated knockdown of *duf*, *rst* or *hbs*, the question arose whether testes muscles are formed correctly despite a reduced nuclei number. We analysed the overall shape and filament arrangement of adult reproductive tracts of RNAi knockdown males using F-actin and DNA staining. The testes of all RNAi knockdown flies had the characteristic coiled morphology of the wild type, and as already mentioned, the adult reproductive tracts did not display any visible defects, constrictions or other alterations compared with the wild type (Fig. 6B, *Dcr-2;duf-RNAi;rst-RNAi*; other knockdown flies not shown). Furthermore, the muscles from *hbs-RNAi*, *duf-RNAi*, *rst-RNAi* and *duf-;rst-RNAi* knockdown males did not display obvious defects. Fibre distribution and filament arrangements of the testes muscle sheaths and the sperm pump musculature were normal when observed under light microscopy (Fig. 7, *Dcr-2;duf*-*RNAi;rst-RNAi*; other knockdown flies not shown) independent of the co-expression of *Dcr-2*. In addition, no defects were found in the muscle sheaths of paragonia, seminal vesicles or ejaculatory ducts of any of the tested RNAi knockdown flies (Fig. S5). Adult males with a reduced Sns level could not be analysed because *sns-RNAi* is pupal lethal.

Our results show that knockdown of IgSF proteins resulted in a reduced nuclei number in testes muscles. Surprisingly, this did not affect (1) the migration of nascent myotubes from the seminal vesicles onto the testes, (2) the morphogenesis of the testes from the larval to the adult shape, (3) filament arrangement and integrity of the reproductive tract musculature and (4) the fertility of the males. Hence, we propose that testes muscles possess mechanisms to compensate reduced fusion.

## DISCUSSION

Despite the differences between the diverse types of striated muscles of *Drosophila*, they share common mechanisms during development. For example, the expression pattern of Duf suggests that Duf plays a similar role in the development of larval muscles (Ruiz-Gómez et al., 2000), leg muscles (Soler et al., 2004), flight muscles (Gildor et al., 2012) and abdominal muscles (Dutta et al., 2004). The cellular adhesion mediated by the IgSF proteins and their signalling in myoblasts is essential for myoblast fusion and formation of striated muscles.

### Smooth-like testes muscles arise from heterotypic fusion of FC-like and FCM-like cells, localized in two distinct myoblast layers

It is unclear how the special smooth-like testes muscles obtain their multinuclear state. In general, cells can fuse or skip cleavage after nuclear division to become multinuclear (Gentric and Desdouets, 2014; Lacroix and Maddox, 2012). It was shown that garland cell nephrocytes can overcome fusion defects by cell division without cytokinesis (Zhuang et al., 2009). In the male reproductive organs, the binuclear epithelial cells of the paragonia arise by cytokinesis skipping (Taniguchi et al., 2014). By contrast, our study demonstrated that multinuclearity in testes muscles is achieved by myoblast fusion. Only one of the nuclei of these small syncytia is positive for *rp298-lacZ*, as we observed previously for the bi-nucleated circular visceral muscles in the embryo (Klapper et al., 2002). We proposed that *rp298-lacZ* – reflecting Duf activity – is no longer transcribed after fusion and thus, no new β-galactosidase is synthesized in the cytoplasm and the protein cannot therefore be imported into the other nuclei of these small syncytia. Thus, downregulation of *duf* transcription could limit the degree of fusion, resulting in small syncytia. By contrast, in the somatic mesoderm, Menon et al. (2005) proposed that reshuffling Duf and its adaptor protein Rols to the membrane provides a mechanism that regulates the rate of fusion to yield larger syncytia in agreement with the observation that all nuclei of the syncytia are positive for rp298 (Menon et al., 2005).

Furthermore, we found that characteristic IgSF molecules of embryonic myoblast fusion were expressed distinctly in these myoblasts. This is in agreement with previous expression patterns of *sns* and *duf* reporter constructs (Susic-Jung et al., 2012). The FCM- and FC-like cell status of two different populations of myoblasts is supported by our finding of only one rp298-positive nucleus in the small syncytia, suggesting that this nucleus of one testis muscle cell originates from a different myoblast to the others.

**Fig. 8.**
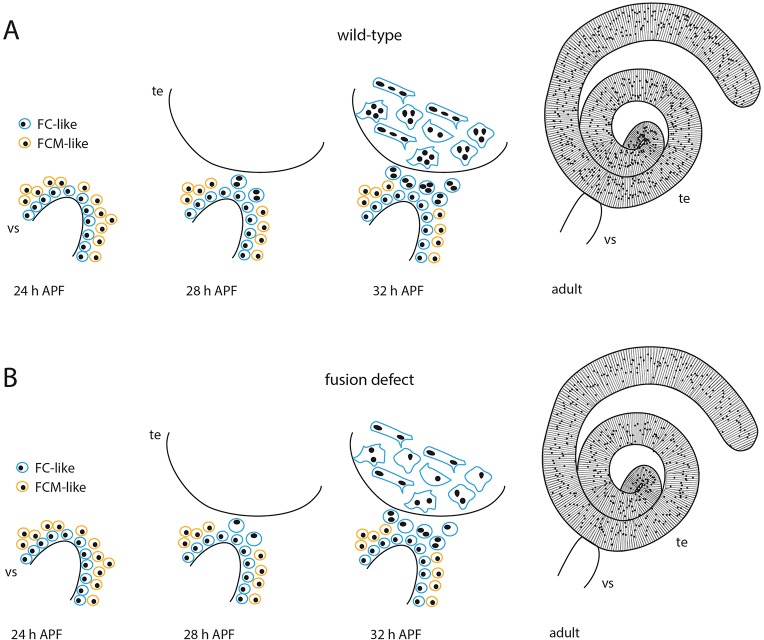
**Scheme of the distinct layers of FC-like and FCM-like cells that fuse on the seminal vesicle to generate the testes muscles.** (A) Myoblasts on the seminal vesicle (vs) are separated into two layers. FC-like myoblasts (blue) express Duf and Rst and lie adjacent to the epithelium of the seminal vesicle, whereas FCM-like cells (yellow) express Sns and appear to be located on the periphery. In heterotypic fusion events, FC- and FCM-like cells fuse to form multinuclear nascent myotubes. These myotubes then migrate onto the testes (te). The adult testis is tightly encircled by multinuclear muscles. (B) The knockdown of IgSF molecules impairs the fusion process. Nascent myotubes with a reduced nuclei number still migrate from the seminal vesicle onto the testes and are able to enclose the adult testes as in the wild type.

Antibody staining revealed that FC-like myoblasts express Duf and Rst and form a basal layer directly adjacent to the epithelium, whereas the FCM-like cells express Sns and lie more in the periphery (schematized in Fig. 8). In the embryo, adhesion molecules are mainly visible at the opposing membranes of growing myotubes and FCMs (Kesper et al., 2007; Rochlin et al., 2010; Sens et al., 2010; Önel et al., 2011). By contrast, testes myoblasts expressed adhesion molecules along their entire surface, which indicates discernible differences in the formation of the different muscle types.

### Duf and Rst might act independently, not redundantly, to create smooth-like myofibres

Knockdown of *duf*, *rst* or *sns* during metamorphosis led to reduced fusion processes in each muscle. Thus, we conclude that myoblast fusion during development of multinuclear smooth-like testes muscles shares common molecular players with previously studied myoblast fusion processes of *Drosophila* and vertebrates. However, we observed a distinct mode of action of Duf and Rst during testes-relevant myoblast fusion. Fusion was also reduced when only *duf* or *rst* was knocked down. In the embryo, only a double knockout leads to defects, whereas the reintroduction of either Duf or Rst is sufficient to rescue the double-mutant phenotype (Ruiz-Gómez et al., 2000; Strünkelnberg et al., 2001). In thoracic muscle development during metamorphosis, *duf*-knockdown myoblasts fuse like the wild type, but *duf-RNAi;rst-RNAi* double-knockdown flight muscles have severe fusion defects, which also indicates a redundant function of Duf and Rst (Gildor et al., 2012). By contrast, Duf and Rst cannot completely substitute for each other in the arrangement of ommatidia during eye formation (Ramos et al., 1993; Reiter et al., 1996). Our data demonstrate that in testes myoblast fusion, the phenotype of the single RNAi knockdown is enhanced when both genes are knocked down simultaneously, which indicates an additive effect.

### Fusion is restricted to the myoblasts at the anterior tip of the seminal vesicles

We observed that testes myoblast fusion was restricted to a small area of the most anterior tip of the prospective seminal vesicles (Figs 2 and 8). Furthermore, our RNAi data indicated that proper expression of Duf, Rst and Sns is crucial for testes myoblast fusion to proceed. As adhesion molecules were expressed in neighbouring FC- and FCM-like cells long before fusion occurs, we conclude that these molecules alone are not sufficient to trigger fusion of adjacent myoblasts. In other fusion processes, key regulators called fusogens are required and are sufficient for fusion to proceed (Aguilar et al., 2013). To date, the fusogen in myoblasts of *Drosophila* has not been identified. Our results demand an accurate spatially and temporally regulated expression of a potential fusogen within the testes myoblasts. Therefore, there might be an extrinsic signal that triggers fusion solely in the myoblasts over the seminal vesicles. Alternatively, fusion might be repressed in all other myoblasts. Clarification of these possibilities will be a focus of future research.

### Compensation of fusion failure leads to correct filament organization of testes muscles, shaping of the testes and male fertility

In *Drosophila* and vertebrates, reduced fusion of striated muscles can lead to severe defects or embryonic lethality (Abmayr and Pavlath, 2012; Rochlin et al., 2010; Önel and Renkawitz-Pohl, 2009). Furthermore, defective morphogenesis of the reproductive system can induce male sterility (Linnemannstons et al., 2014). However, despite a reduction in fusion in the knockdown testes muscles, no obvious defects in formation of the reproductive tract and progression of spermatogenesis were detected in the resulting adult males. The seminal vesicles were always connected to the testes, and the myotubes migrated onto the testes. Even the filament arrangement in these flies appeared normal in light microscopy, independent of the level of the fusion block (Fig. 7, Fig. 8B). Thus, we assume that testes muscles remain functional even if fusion is reduced. We conclude that progression of myoblast fusion is not required for further development of the testes muscles, including attachment, filament production and arrangement. Kreisköther et al. (2006) showed that sarcomeres of striated larval muscles are established very late in embryogenesis after muscles attached to the epidermis. This process seems to proceed independent of myoblast fusion in some muscles also in fusion mutants (Drysdale et al., 1993). Similar observations have been reported for striated muscles in zebrafish; mutants of the IgSF genes *jamb* and *jamc* display fusion defects during fast-twitch muscle development, whereas elongation and sarcomere formation are not disturbed (Powell and Wright, 2011).

In summary, our results indicate that testes muscles compensate for fusion defects in a manner not previously reported. We suggest that testes muscles have an enormous potential for increasing the plasticity of smooth muscles. We propose that this plasticity compensates for the lack of satellite cells in *Drosophila* and their ability to regenerate muscle defects and thereby safeguards reproductive capability.

## MATERIALS AND METHODS

### Fly stocks

Flies were maintained and RNAi was performed in *Drosophila* standard medium at 25°C. *w^1118^* (BL6326) was used as the wild-type control. The following transgenic flies were used: *rp298-lacZ* (Nose et al., 1998), *Mef2-Gal4* (Ranganayakulu et al., 1995), *UAS-Dcr-2;Mef2-Gal4* (BL25756), *UAS-mCD8-GFP* (BL32186), *UAS-duf-RNAi* (V3111), *UAS-rst-RNAi* (V951), *UAS-hbs-RNAi* (V40898) and *UAS-sns-RNAi* (V108577). It has been shown previously that these RNAi constructs mediate efficient knockdown with other driver lines (Gildor et al., 2012; Machado et al., 2011; Susic-Jung et al., 2012). BL flies were obtained from Bloomington *Drosophila* Stock Center; V flies were ordered from the Vienna *Drosophila* RNAi Center.

### Tissue preparation and immunofluorescence

To acquire pupae of a defined age, white prepupae were collected at 0 h APF and aged on a moistened filter. Pupae or adult flies were dissected in a drop of phosphate-buffered saline (PBS; 0.13 mM NaCl, 7 mM Na_2_HPO_4_, 3 mM NaH_2_PO_4_) under a stereomicroscope. For antibody staining, tissues were fixed in 3.7% formaldehyde in PBS for 20 min, primary antibody was incubated overnight at 4°C and secondary antibody was added to the specimens at room temperature for 1 h. Hoechst 33258 (3 µg/ml; Sigma-Aldrich, 94403) was used to label DNA and Atto565-phalloidin (4 nmol/l; Sigma-Aldrich, 94072) was used to stain F-actin.

The following antibodies were used: anti-GFP (1:2000; Abcam, ab6556), anti-tropomyosin (1:1000; Abcam, ab50567), anti-phosphorylated histone H3 (Ser10) mitosis marker (1:500; Millipore, 06-570), anti-Mef2 (1:500; kindly provided by Hanh T. Nguyen, University of Erlangen, Germany), anti-Duf and anti-Rst (1:500 and 1:50, respectively; both gifts from Karl-Friedrich Fischbach, University of Freiburg, Germany), anti-β-Gal (1:1000; Biotrend, RGA1-45A-Z) and anti-Sns (1:250; generated by Pineda Antibody Service, Berlin). For fluorescent immunohistochemistry, the following secondary antibodies were used: anti-rat Alexa Fluor 488 (Jackson ImmunoResearch Laboratories), anti-rabbit DyLight488 (Vector Laboratories) and anti-guinea pig Cy2 (Jackson ImmunoResearch Laboratories).

### EdU labelling of pupal tissue

To detect proliferation, Click-iT EdU Alexa Fluor 594 (Molecular Probes, Invitrogen) was used. EdU (0.1 µl of 0.5 mM) with Toluidine Blue (0.25%), for a better visualization in PBS, was injected into the dorso-medial part of the pupal thorax as described elsewhere (Ito and Hotta, 1992) at 24 h APF and incubated for 8 h at 25°C. After dissection and fixation of the testes, anti-GFP staining was performed. EdU was detected following the supplier's instructions.

### RNA isolation from myoblasts

Cells were isolated following the protocol of Wang et al. (2006) with modifications. Briefly, 20 *UAS-mCD8-GFP;Mef2-Gal4* genital discs of 8, 16, 24 and 30 h APF were dissected in cold dissociation buffer (Sigma, C1544) and kept cold until further processing. Washed genital discs were then dissociated by elastase treatment (5 µg/ml; Sigma, E0127). Myoblasts were purified using magnetic beads [Dynabeads Mouse CD8 (Lyt2), Invitrogen] according to the supplier's instructions. Subsequently, total RNA was isolated from these myoblasts using the RNAqueous-Micro total RNA Isolation Kit (Ambion) following instructions therein. RT-PCR was performed using the OneStep RT-PCR Kit (Qiagen) and oligonucleotides listed in Table S3.

### Image acquisition and processing

Conventional fluorescent images and optical sections were gathered with a Zeiss AxioObserver Z.1 inverse microscope with an attached ApoTome that was used for structured illumination microscopy. Images were merged using Adobe Photoshop CS 5.1, plates were arranged in GIMP 2.8 and charts were generated in Microsoft Excel 2010.
